# To challenge oneself as a childbearing woman—the lived experience of vaginal birth after caesarean section in Sweden

**DOI:** 10.1080/17482631.2019.1605784

**Published:** 2019-05-03

**Authors:** Ida Lyckestam Thelin, Ingela Lundgren, Christina Nilsson

**Affiliations:** a Institute of Health and Care Sciences, The Sahlgrenska Academy at the University of Gothenburg, Gothenburg, Sweden; b Department of Obstetrics and Gynaecology, Sahlgrenska University Hospital, Gothenburg, Sweden; c Faculty of Caring Science, Work Life and Social Welfare, University of Borås, Borås, Sweden

**Keywords:** Women, childbirth, VBAC, phenomenology, experiences, caesarean section

## Abstract

**Purpose:** There is a need to gain deeper knowledge about women’s experience of vaginal birth after caesarean section (VBAC). Considerable research has been conducted on VBAC; however, only a few qualitative studies focus on women’s experiences. Therefore, the aim of this study was to describe the lived experiences of VBAC among women resident in Sweden, a country with a high VBAC rate.

**Method:** This studywas performed in accordance with a phenomenological reflective lifeworld approach. Interviews were conducted with nine women in an urban region of Sweden one year after their VBAC birth.

**Results:** The essential meaning of the studied phenomenon is *“to challenge oneself as a childbearing woman”*, which is further described by its four constituents: “striving for support from professionals”, “desiring the experience”, “contrasting and comparing memories of two different births” and “being part of the birthing culture”.

**Conclusions:** The experience of VBAC meant regained trust in the ability to give birth vaginally. The women lacked follow-up and support after the caesarean section (CS), during the subsequent pregnancy and the forthcoming VBAC. Enhanced support could be a key factor in helping women meeting the challenge and feel confident about giving birth vaginally despite their previous experiences of CS.

**Abbreviations:** VBAC: vaginal birth after caesarean section; CS: caesarean section

## Background

Labour and birth, is an important life event (Larkin, Begley, & Devane, 2009) and are considered to be one of the most significant experience in a woman’s life (Downe, 2008). Nevertheless, childbirth experience is multi-dimensional, complex and unique to each woman (Larkin et al., 2009). A woman’s experience of her first birth is of importance for her attitude towards how she wishes to give birth in the future. A positive birth experience may be strengthening for the woman’s self-confidence (Simkin, 1996). However, negative birth experiences, can have a short and long-term impact on the women’s health and well-being (Garthus-Niegel, Soest, Vollrath, & Eberhard-Gran, 2013; Simkin, 1991; Waldenström, Hildingsson, Rubertsson, & Rådestad, 2004). If the woman has a negative experience she might feel disconnected, helpless and have felt that her body failed her (Rijnders et al., 2008; Waldenström et al., 2004). The risk for a negative birth experience increases if the birth ends up with an emergency caesarean section (CS) (Waldenström et al., 2004).

The rising rate of CS globally is of general concern because of the higher risks for women’s health and well-being even though the rates vary considerably in different countries (EURO-PERISTAT, 2013). The reason for the rising CS rate is multifactorial; and interventions to reduce medical complications among women and their babies have shown little effect (Betrán et al., 2018). However, one common reason for performing a CS is that the women have undergone a previous CS (Poignant, Hjelmstedt, & Ekeus, 2012).

Due to the rising rate of CS, the women with previous CS, and health professionals need to consider the mode of birth in the subsequent pregnancy. Similar to the CS rate, the vaginal birth after caesarean (VBAC) rate differs across maternity settings and countries. In Europe, VBAC rates vary widely and have declined considerably in recent years, especially in Spain and Portugal, where the rates are around 20–30%. These rates are significantly lower than in Sweden, Finland and the Netherlands where rates of VBAC are between 45% and 55% (EURO-PERISTAT, 2008).

The decision to plan for a VBAC should be individually based with the considerations that recent research has shown that VBAC is recommended as safe and best practice for most women with a previous CS and their babies (ACOG, 2017). Even if a vaginal birth is the safest option for most women, a negative birth experience may influence the women to request a planned CS in subsequent pregnancies (Karlstrom et al., 2010). Negative birth experiences in women are often associated with emergency CS (Nystedt & Hildingsson, 2018) and vacuum extraction (Smarandache, Kim, Bohr, & Tamim, 2016). However, an uncomplicated vaginal birth can also be experienced as negative by women, and such experiences are often related to a lack of care with feelings of loneliness and being out of control (Elmir, Schmied, Wilkes, & Jackson, 2010). Consequently, fear of childbirth after a negative birth experience is of importance (Dencker et al., 2018; Nilsson et al., 2018) and can be a reason for requesting a CS in the next pregnancy (Karlstrom et al., 2010; OECD, 2017).

Considerable research has been conducted on VBAC; however, only a few qualitative studies focus on women’s experiences. In a meta-synthesis of women’s experiences of VBAC, few studies were identified for inclusion (Lundgren, Begley, Gross, & Bondas, 2012). These studies were limited to a small number of Anglo-American countries, mainly, with low VBAC rates. The results revealed that the women felt that they were “grouping through the fog”, meaning that they searched but received insufficient information about VBAC (Lundgren et al., 2012). In countries with a low rate of VBAC, some studies showed that health professionals did not provide women with enough evidence-based information on the risks and benefits of VBAC to support their decision-making (Lundgren et al., 2012; Nilsson et al., 2017). Studies from these countries revealed that women felt uncertain in their decision regarding mode of birth following a CS (Farnworth, Robson, Thomson, Watson, & Murtagh, 2008; Frost, Shaw, Montgomery, & Murphy, 2009; Guise et al., 2010; Moffat et al., 2007). Moreover, studies focusing on women’s experiences of VBAC in countries with low rates of VBAC have highlighted the women’s belief in the importance of having a natural birth (Meddings, Phipps, Haith-Cooper, & Haigh, 2007; Wang, Chung, Sung, & Wu, 2006) and that they associate vaginal birth with several positive aspects, such as a strong sense of responsibility for giving birth (Lundgren et al., 2012). VBAC is regarded as good for the baby and for the relationship between mother and baby as well as a meaningful and strengthening experience for the women (Lundgren et al., 2012).

To our knowledge, there is only one focus group study conducted with the aim to explore women’s views in countries with high VBAC rates (Nilsson, van Limbeek, Vehvilainen-Julkunen, & Lundgren, 2015). The results revealed that the women wanted to receive individual information about VBAC and support from a midwife and/or obstetrician during pregnancy and birth (Nilsson et al., 2015).

In summary, there is limited qualitative research on women’s lived experiences of VBAC, in countries where VBAC is common. Therefore, it is important to gain a deeper understanding of women’s experiences in these countries, that can be used to inform interventions that support VBAC in women, but also to improve maternity care for women with previous CS. The aim of this study was to describe women’s lived experiences of VBAC in one region of Sweden, a country where VBAC is common.

## Method and approach

In order to describe the chosen phenomenon; women’s lived experiences of VBAC, a reflective lifeworld approach was used (Dahlberg, Dahlberg, & Nyström, 2008). This approach, which is based on the phenomenological philosophy of Husserl (1859–1938) and Merleau-Ponty (1908–1961), is well suited to describe and understand complex human experiences, particularly in healthcare settings (Dahlberg et al., 2008). Essential concepts in the phenomenological tradition are intentionality and bracketing. Intentionality is described by Husserl as the focus of consciousness, i.e., the ability to direct attention towards objects, and how it is experienced (Bengtsson, 1995). Bracketing was introduced by Husserl with the attempt to restrain old past knowledge about a research phenomenon so that researcher’s attention was directed only on the present phenomenon without preunderstanding (Bengtsson, 1995).

The reflective lifeworld approach by Dahlberg et al. (2008) was used to illuminate the essential meaning of the phenomenon and its variations, and thereby develop the understanding for women’s lived experiences of vaginal birth after a previous CS. This approach and the entire research process are characterized by the phenomenological attitude, which means that the researcher stays open for the phenomenon, including a self-reflective attitude (Dahlberg, 2001; Dahlberg, Gjengedal, & Råheim, 2010).

### Settings and participants

Maternity care in Sweden is free of charge and funded by taxes, with almost all births taking place in hospitals. Homebirth is generally not included in the healthcare system with two exceptions: the districts of Stockholm and Västernorrland. Midwives in Sweden have independent professional responsibility for normal pregnancy and childbirth, including the responsibility for consulting other health professionals when complications occur (SBF, 2018). In the event of the latter, the obstetrician is responsible, but the midwives remain involved in the woman’s care. Due to Swedish health-care laws, women in Sweden are not entitled to have a CS without a medical reason (Wiklund, Andolf, Lilja, & Hildingsson, 2012). Nevertheless, a CS can be performed if the woman suffers from fear of childbirth, provided that the individual circumstances are discussed with the obstetrician and that the woman has received information about the risks associated with CS along with supportive counselling at specialized “fear of birth” clinics (Wiklund et al., 2012). Approximately 8% of the total number of CS (17.6%) in Sweden are performed due to the women’s request and one important reason for this preference is fear of childbirth (Karlstrom et al., 2010; Socialstyrelsen, 2017; Wiklund et al., 2012).

Sweden has no national guidelines regarding counselling and care for women with a planned VBAC, but there are local guidelines (Lundgren, van Limbeek, Vehvilainen-Julkunen, & Nilsson, 2015; Nilsson et al., 2015). Generally, women who have undergone a previous CS without complications are advised to plan for a VBAC. Midwives provide care for these women during pregnancy, labour and birth and consult the obstetrician when required.

The women in the present study experienced their VBAC at a university hospital in the southwestern part of Sweden, where the three labour wards combined had approximately 10,100 births annually during 2013 and 2014. The inclusion criteria for the study were Swedish-speaking women who had undergone a VBAC in the previous year. Information about women who had a CS birth with their first baby followed by a spontaneous vaginal birth with their second baby was obtained from the hospital medical record system in 2013 and 2014. Potentially eligible women were consecutively identified. Women with psychiatric disease or distress, drug abuse, had experienced intrauterine foetal death, neonatal death or if the neonate was severe ill at birth were excluded from being selected. In order to capture as many variations of the phenomenon as possible, age and reason for the previous CS (planned or emergency) were taken into account. Potential participants were informed about the study by the researcher (ILT), first by letter and then orally over the phone a week after receipt of the letter. A sample size for the study was not predetermined; rather the intention was to have sufficient participation to ensure varied and rich data from each informant (Dahlberg et al., 2008). Of 14 women who were contacted, five declined to participate due to lack of time and/or lack of childcare. Eventually, nine women, aged between 26 and 42 years, who had experienced a VBAC with their second baby, participated in this study. The participants were educated to at least upper secondary school, and seven of them were educated to university level. At the time of the interviews, all of the participants were employed and were reportedly healthy. Table I presents additional information about the participants.

**Table I.** Description of participants.

Interview Person	Age when interview was conducted	Wanted a vaginal birth with the first child	Fear of childbirth during first or second pregnancy	Indication for the previous CS^1^	Elective or emergency CS	Wanted to have a VBAC^2^	Year when having the VBAC
1	26	Yes	No	Breech	Elective	Yes	2012
2	37	Yes	No	Dystocia	Emergency	Yes	2012
3	34	Yes	No	Breech	Elective	Yes	2012
4	33	Yes	No	Dystocia	Emergency	Yes	2012
5	37	Yes	No	Dystocia	Emergency	Yes	2012
6	37	Yes	No	Dystocia/Asphyxia	Emergency	Yes	2013
7	38	Yes	No	Severe preeclampsia	Emergency	Yes, but not if being induced again	2013
8	31	Yes	No	Undiagnosed breech	Emergency	Yes	2013
9	42	Yes	No	PROM^c^ and breech	Emergency	Yes	2013

^a^Caesarean section

^b^Vaginal birth after caesarean

**^c^**Premature rupture of the membranes

### Data collection

Interviews were conducted with the women approximately one year after their VBAC. Each woman was interviewed on one occasion. The women were asked to select the location for the interview. Six interviews were conducted in a quiet room at the University of Gothenburg and three in the homes of the women. The interviews started with an open question: “Can you tell me about your experience of giving birth vaginally after your previous CS?”. When appropriate, additional questions were posed to obtain a deeper understanding of the women’s experiences. These questions included “What did you think about that?”, “What do you mean?”, “Can you give an example?” and “How did that make you feel?” Once the interview was over, the researcher sought additional information from each woman to provide some background details and a description of the sample. This information included the women’s ages, if they wished for a vaginal birth with their first child, whether or not they experienced fear of childbirth, the indication for the CS, if the CS was an elective or an emergency CS, if they wanted a VBAC and the year they had the VBAC. Data collection took place from April 2013 to October 2014 and the interviews lasted between 25 and 60 min.

### Data analysis

The data analysis was performed in accordance with a reflective lifeworld approach as described by Dahlberg et al. (2008) and followed a movement between the whole and the parts. The interviews were transcribed verbatim by the first author (ILT). Each interview was read repeatedly in order to gain a sense of the whole. Thereafter, the text was read with the phenomenon in mind, and changes in meaning with respect to the phenomenon “women’s experience of VBAC”, was marked in the text and described with a few words (Dahlberg et al., 2008). In order to structure these meaning units, different clusters were formed. Each cluster consisted of meaning units related to each other. Clusters can be described as a framework for temporary patterns of meanings that helps the researcher to see structures that give a picture of the essential meaning (Dahlberg, 2011). When all meanings had been identified and organized in the different groups of clusters, the clusters were read and re-read with the phenomenon in mind. In this phase of the analysis, the movement between the whole and the parts of the data was used together with a movement between figure and background (Dahlberg et al., 2008). To work in terms of figure and background means of pick up clusters of meaning and watching it as a figure against the others as background, and so on. The aim was to discover a pattern of meanings. (Dahlberg, 2011). Thereafter, the phenomenon’s essential meaning was obtained and formulated. An essential meaning can be described as an abstraction of the phenomenon’s general and invariant meaning. The essential meaning is further described by its constituents, i.e., the variations of the essential meaning. Together they constitute the essential meaning in a more contextual way (Dahlberg et al., 2008).

The analysis was done together with co-authors. During the data analysis process, the researchers strived for having a reflective phenomenological attitude toward the data. This reflecting attitude is described by Dahlberg et al. (2008) as bridling, which means to have a respectful reflective attitude and actively but patiently wait for the phenomenon and its meanings to appear. Dahlberg et al. (2008) describe that the term bridling within reflective lifeworld research, relates to Husserl’s “bracketing”, i.e. the restraining or parenthesizing of one’s pre-understanding of the studied phenomenon. In contrast to bracketing, bridling can be understood as a constant questioning of the presupposed assumptions that the researcher has in relation to the phenomenon being studied (Dahlberg et al., 2008). Furthermore, Dahlberg et al. (2008) argue that bridling focus on the whole event of understanding and is pointing forward. While bracketing is directed backwards, fighting pre-understanding and keeping it in check “back there”, not letting it affect what is happening here and now (Dahlberg et al., 2008).

In this study, bridling was done by reflecting on the pre-understanding of the phenomenon before entering the texts and during the entire process. Moreover, the attempt to remain in the analysis phase, i.e., reading the texts over and over to gain a sense of the whole, to stay close to the data and constantly move back and forth between the whole—to the parts—and the whole.

### Ethical considerations and approval

Ethical approval was obtained from the Regional Ethical Review Board in Gothenburg, ref.no. 155–13 and was performed in accordance with the ethical standards as described in the Declaration of Helsinki. The participants provided written, informed consent to take part in the study and were aware of the guarantee of confidentiality and their right to withdraw at any time before, during or after the interview. They all permitted the interviews to be recorded and gave consent to publish the result in a scientific journal.

## Findings

The essential meaning of VBAC as experienced by women who had undergone VBAC was described as “*To challenge oneself as a childbearing woman”*. Both the CS and the VBAC existed in parallel, yet different stories and remained with the women as intertwined memories. The different childbirth experiences were not separated, rather they coexisted. *To challenge oneself* meant not relinquishing the expectation of a vaginal birth during the second pregnancy despite previous experiences. This was associated with multiple challenges in relation to the CS, feelings about oneself, and the body. The previous CS contributed to insecurity about chances of a successful VBAC. To meet the challenge involved, the women required professional support and confirmation of their ability to give birth vaginally. Achieving VBAC was nourished by a strong force, a determination and an inherent belief of giving birth vaginally. During the second pregnancy, the scar from the CS was a constant reminder of the previous birth, which created uncertainty. The women strived for an identity *as a childbearing woman* by meeting the challenge of VBAC. When having experienced the VBAC, the self-image was changed and enabled the women to feel capable of giving birth and regain confidence in their body.

The essential meaning is described below from its four constituents: “striving for support from professionals”, “desiring the experience”, “contrasting and comparing memories of two different births” and “being part of the birthing culture”.

### Striving for support from professionals

The women needed support to work through their experiences of the previous CS when the second pregnancy was confirmed. Memories and emotions surfaced that led to a deep need to understand why a CS was performed during the first birth, particularly if the reason had not been clearly identified. However, they experienced resistance from midwives and obstetricians in discussing their previous CS, which evoked feelings of being alone.

*I wished that the midwife had said that I could meet someone to talk it through. I did not have any support during the pregnancy with my second child*. (Woman 7)

When the second birth was approaching, the women described an urge to talk about both the CS and the forthcoming VBAC. Some of the women were referred by their midwife to the Aurora team who run a special “fear of birth clinic” for women who are experiencing fear of childbirth. This referral, however, was not always desirable to the women as they thought it could stigmatize them as having a fear of childbirth. In their opinion, talking to their midwife who cared for them during the whole pregnancy would have been more “natural” and convenient. Despite finding the counselling provided by the Aurora team helpful, they considered it inconvenient due to the different care setting.

*It felt like the midwife was almost was afraid to talk about it [the CS and the forthcoming VBAC]. She said that it was better to talk to the Aurora team. I think that my midwife would have been able to help me*. (Woman 5)

The women felt that they had fewer visits with the midwife during the second pregnancy compared with the first. A desire for more time with the midwife was described, and lack of time contributed to feelings of being unable to discuss reflections and questions. The women considered that the focus of the visits was primarily on their own physical status and that of their unborn babies.

*The midwife did listen when I was there, but there were few visits, especially with the second child, and it felt like there were things that I did not have time to talk through properly*. (Woman 8)

Furthermore, the women desired support in the process of preparing themselves for the VBAC. Their preparations were driven by a strong inner desire to give birth vaginally. The women had different ways of preparing for this based on their experiences of the previous birth. For example, they discussed options for pain relief, and raised concerns about overuse of anaesthesia which can cause prolonged labour. Some of the women wished for their second labour to be managed differently than their first and expressed a desire to have help with writing a birth plan to ensure this would be the case. This plan was seen as an assurance that in the event of a lack of progress, a CS would be performed earlier than was the case during the first labour.

*I expressed myself clearly during the second pregnancy that I didn’t want them to handle it the same way [as the first labour]… so I had to see a doctor, and I thought that the idea was that he would write some kind of birth plan. If the same scenario occurred again [prolonged labour], they should not wait as long [as with the first CS], but I felt it was quite a meaningless conversation. The doctor said that he could not write a plan for the obstetrician at the hospital, so I still felt that what might happen was left to the moment*. (Woman 4)

The women felt that they were regarded as experienced by the midwives, and not considered in need of additional information because they were mothers. The women could not remember all of the information about relaxation, breathing techniques and different pain relief methods that they had received during the first pregnancy. This contributed to stress and a sense that they should prepare themselves; however, they also expressed that there was not enough time for this during the second pregnancy because they were preoccupied with caring for the first child in combination with their return to work after parental leave. Some of the women did not consider that they might need any special preparation as they expected to be given any information they required once they arrived at the hospital.

*I did not prepare myself very much before the vaginal birth, even though I constantly thought that I should prepare myself…When I had been [for a visit] at the hospital I felt that there were people there who would help me when I needed it and that I didn’t have to prepare myself. I felt very relieved*. (Woman 8)

The women mentioned various reasons for actively striving for support from the midwives and the obstetricians to enable them to have a vaginal birth instead of a repeat CS. Some of the women had been very unwell and tired after the surgery and a few had long-term symptoms related to the scar, such as sensory loss, a tight feeling and impaired physical appearance. The women also expressed concern about being unable to care for their first child in the event of a repeat CS.

*It’s a bigger deal having a CS; it’s a big operation that makes you weak and you can’t get out of bed immediately afterwards and I was tied to the bed with all these tubes and everything, so I could not really hold the baby*. (Woman 3)

The women also desired help in managing the fear of potential complications associated with the previous CS. They were afraid that a repeat CS would have an impact on the number of children they would be able to have in the future and that the scar from the previous surgery would rupture during pregnancy or birth. The women also expressed concerns about never being able to experience a VBAC in the event of a second CS.

*My concern was that if I had a repeat CS, it would limit the number of children I might have in the future*. (Woman 6)

After the CS, the women received diverging information about how long they should wait before becoming pregnant again, which varied from six to 18 months. During the second pregnancy, this led to concerns about having become pregnant too soon, thereby exposing themselves and their child to increased risk. Some of the women who received no support in dealing with their concerns frequently searched for information about VBAC online. The information found on the internet often highlighted the risks associated with VBAC. However, this negative information did not influence the decision to give birth vaginally the second time.

*I did not feel less anxious after reading the information on the internet about VBAC; it was rather the other way around. I felt that the online information about VBAC was mostly negative*. (Woman 9)

### Desiring the experience

The women desired different labour and birth than before. Using the previous CS as a reference and strategy for changing their own as well as the clinicians’ behaviour during the next birth was repeatedly mentioned. The women were more focused on the task ahead and demanded more of themselves and of the midwives and the obstetricians to avoid that the second birth being similar to the first; for example, becoming prolonged or ending in a CS birth. Memories of the first labour and birth motivated women to be more active during the subsequent labour to achieve normal labour progress.

*I know I was in a different position [during the second labour] and in retrospect I think that I wanted the child to come down properly. I had talked extensively with the midwives, and it was stated in my medical record that I wanted to give birth vaginally*…*I had thought a lot about what went wrong [during the first labour], so I decided not to take the epidural because I was convinced that the epidural made the contractions stop and was the reason the first labour ended with a CS*. (Woman 6)

The women took greater responsibility themselves during the second labour and birth rather than depending solely on the midwives and/or the obstetricians to decide what was best. This, in combination with the preparation that was undertaken during the second pregnancy, gave the women an inner sense of security. The ambition was clear, the labour should be successful. Although the women felt an inner assurance that they would succeed with the VBAC, support from the midwives and the obstetricians was important. A lack of such support, for example, when the women felt their painful contractions were not being taken seriously because they were being viewed as “obstetric first-time mothers”, was a negative experience for them.

*So that was the only thing I felt was a bit negative. When I arrived [at the hospital] no one thought that I was as close to birth as I was. Everyone just said: “relax, take it easy”, but I felt that it was approaching*. (Woman 3)

Although the women were confident it was evident that the midwife’s attitude and approach were of great importance. A skilled midwife was described as someone who appeared to know what she was doing, was considerate and guided the women through the various stages of labour. When the midwife clearly stated that she had an action plan to prevent another CS, the women felt secure.

*They [the midwives] told us the plan and I appreciated that. It didn’t seem like something that was just scribbled down. It was more like: “if nothing has happened at six o’clock we may break the membranes”… They really described it in detail*. (Woman 4)

One woman described her midwife as not supporting her at all during the VBAC, with the result that it became a painful and difficult experience in which she felt abandoned and deceived, albeit this time, not by her own body. The midwife’s unsupportive attitude resulted in the woman perceiving herself as a difficult patient. Although she spent a great deal of time attempting to understand and find explanations for the midwife’s cold and harsh attitude, she found it difficult to reconcile herself with the experience.

*The midwife made me feel that I was annoying and awkward; talked behind my back and over my head. I felt like a very difficult patient…the collaboration with the midwife [during the VBAC] wasn’t good, and I thought that overall, the whole experience was worse than the CS*. (Woman 2)

### Contrasting and comparing memories of two different births

During the VBAC, feelings of being both strong and weak occurred. The vaginal birth was experienced as a gratifying confirmation that the body functioned and as having strengthened them, which was not the case with CS. It was invigorating to give birth to the baby by their own effort, thus the vaginal birth was associated with what it means to be a woman. Although the women stressed that their feelings of happiness and joy were similar when the baby was delivered by CS, these feelings were more powerful after the vaginal birth. The vaginal birth resulted in energy and a feeling of being powerful, strong and euphoric and seemed to improve their ability to handle sleep loss immediately afterbirth.

*After [the VBAC], I had very strong feelings similar to an endorphin kick. I just sat there: “Ah!” … You feel great and there was a lot of energy, and then you don’t need to sleep so much, you actually manage the first period when the baby is newborn better in that sense*. (Woman 5)

Most of the women experienced vaginal birth as a quick process. This was something unexpected because they were aware that it could take time due to their previous experience of prolonged and obstructed labour. A quick birth was described as easy in retrospect. It also emerged that even the midwives had not thought that the women would give birth so quickly due to the previous CS.

*And then it went fast, so after like an hour or so, from six to ten centimeters, it took only twenty minutes, and then I was fully dilated and gave birth*. (Woman 1)

Few of the women described having a similar course of labour, with their VBAC labour, to that of their previous labour whereas others described having a completely different experience. They found it difficult to describe the sensation of contractions on the body, as they had never previously experienced such pain.

*But still it is strange that you want to experience it again, so it couldn’t have hurt that badly*…*But it is difficult [to describe the experience of having contractions]. Just as the contractions were coming, it did hurt very badly of course, but you forget*. (Woman 1)

The women described not being separated from their babies directly after the VBAC as being different from that which happened after the CS. Being able to hold the baby to one’s breast after giving birth, relax, recover and just enjoy being a family and starting to connect with the baby immediately were described as something natural and amazing. The women also stated that holding the baby in their arms gave them a sense of being capable, which had been difficult after the CS due to nausea, pain and reduced ability to move.

*I thought that it was so nice after she was born. She was together with us and I didn’t have to leave her which was the case after the CS*. (Woman 3)

Another important difference between the two modes of birth was the subsequent healing process. Although some women described pain after the vaginal birth, especially pain associated with having sutures in relation to the suturing, it was not comparable to the pain and the healing process that they experienced after the CS, where the surgical scar caused discomfort for a long time. Due to the impression of recovering more rapidly than after the CS, several women felt that the vaginal birth passed almost unnoticed, as if they had not given birth, due to feelings of returning to themselves sooner than they did after the CS. It was also described as a quick return to normality, as they could get up and move around almost as usual afterwards.

*I was alert and capable of getting up and walking immediately after [the vaginal birth]. It felt hardly as if I had given birth*. (Woman 5)

Thoughts on whether the mode of birth impacted on the women’s relationship with their first and second baby differed between women. Some experienced it was easier to connect with the second child.

*I found it much easier to connect with the second child and there’s still a big difference. I don’t know if the problem with our relationship [with the first child] depends on the mode of birth or whether it was down to the fact that more comprehensive life adjustment was necessary after the first child*. (Woman 8)

The women reflected upon whether the different modes of birth might have influenced their babies, and if so, in what way. A happy baby resulted in a more positive perception of the birth, overall, while some perceived that their relationship with the first and second baby was the same. The woman whose VBAC was a negative experience described how it took time for her to connect with her baby, which caused a sense of strain and emptiness.

*I could not look at my child or connect with him. I was just shivering and closed my eyes. I was completely inside myself*. (Woman 2)

### Being part of the birthing culture

VBAC resulted in feelings of being part of a birthing culture. In this context, vaginal birth is considered the norm by family, friends, acquaintances, clinicians (midwives and obstetricians) and the community. The culture itself is demonstrated by the fact that both the women and the clinicians considered vaginal birth to be the norm, even after one CS. This was also evident in the disappointment that the women felt when they were prepared to give birth vaginally to their first baby but were unable to do so.

*I don’t know [where the attitude to vaginal birth comes from]. I was just so set on it, and there is not much talk about CS in parental education [during the first pregnancy], or maybe I didn’t listen because I thought that it didn’t apply to me. So, I was not at all prepared for a CS*. (Woman 4)

Furthermore, neglecting to follow up the women after the CS and failing to address the women’s need to talk about both the CS and the planned VBAC during the second pregnancy also highlights clinicians’ attitudes in the existing birthing culture.

It was important for the women that the second pregnancy was treated normally, but they wanted more information about VBAC. The VBAC gave the women an opportunity to be part of the birthing culture and identify themselves with giving birth in the same way as most women do. A vaginal birth meant giving birth normally without surgical assistance; a view which was strengthened by women in their environment who expressed that having a child by means of a CS was not a true birth.

*I’m just happy that I was being able to experience it [vaginal birth], actually. If I had two children by CS, I would not have known what I had missed*. (Woman 4)

Despite the difficulties describing their exact emotions, a vaginal birth was associated with being a woman and provided a real sense of giving birth to the baby themselves. While the women expressed gratitude that their bodies could manage a vaginal birth, they were reluctant to judge which mode of birth should be preferable for other women. Nevertheless, they emphasized the benefits of vaginal birth, such as being better for the child, not affecting themselves physically to the same extent as a CS, eliminating the long recovery after a CS and being able to connect with their newborn babies almost immediately after the birth.

*It must be better for the child to be born naturally than just being lifted out like that*. (Woman 3)

## Discussion

This study focuses on women’s lived experience of vaginal birth after CS, a phenomenon rarely described in other studies from the present context. To our knowledge, this is the first study with individual interviews from a country where women are expected to give birth vaginally even with a previous CS. The essential meaning “to challenge oneself as a childbearing woman” indicates a strong desire to give birth vaginally and relates to the identity as a childbearing woman. Even if the CS scar caused an uncertainty in the challenge and therefore, to meet this challenge the women needed an inner determination, as well as support. When having experienced the VBAC, the women described that they could identify themselves with other childbearing women who have given birth vaginally. However, the women had to struggle to get the support they needed. One important finding for maternity care professionals is that leaving these women without additional individual support can evoke feelings of loneliness and despair. The dilemma seems to exist in the balance of the women’s needs and the existing care system. Women expecting their second child are regarded as experienced when it comes to childbirth. Therefore, it is crucial for these women that the focus is on their individual need of support and information regarding both their previous experience of CS and the forthcoming VBAC to increase health and well-being among these women.

The strong desire for VBAC among the women is also described in a study from Australia (Phillips, McGrath, & Vaughan, 2009), in which the women expressed that they desperately wanted a VBAC because of the physiological and psychological benefits for both themselves and their babies. Although it was described by the woman in our study as an overwhelming feeling to become a mother, the VBAC meant something different to the women than the previous CS. This can be compared with the results in a study from Australia (Fenwick, Gamble, & Hauck, 2007), the women believed that vaginal birth was a part of being a woman and mother. They also highlighted the benefits of vaginal birth compared to CS, enhancing the health and well-being of both mother and baby, promoting maternal-infant relationship and easing the transition into motherhood (Fenwick et al., 2007).

The women in our study actively sought support and information from the midwives, and the obstetricians when their second pregnancy was confirmed, a finding also presented in other studies (Dahlen & Homer, 2013; Godden, Hauck, Hardwick, & Bayes, 2012). However, the women felt that both midwives and physicians were reluctant to discuss their previous birth experience. This finding probably reflects current routines in Sweden, where it is common to refer women to special fear of birth clinics if the midwife or physician considers any anxiety in the woman (Larsson, Karlström, Rubertsson, & Hildingsson, 2015; Wulcan & Nilsson, 2019). This routine caused distress for some of the women in our study as they were concerned that they might be labelled as having fear for a vaginal birth or having psychological issues, when this was not the case. According to our study, we suggest that midwives who are caring for women during pregnancy develop their ability to support these women before referring to special clinics. However, for some women who experience intense fear of childbirth after a CS, counselling in such clinics has shown to be beneficial (Larsson, Hildingsson, Ternström, Rubertsson, & Karlström, 2018; Larsson et al., 2015; Ryding, Persson, Onell, & Kvist, 2003).

Midwives and obstetricians working in Swedish maternity care consider pregnant women who have undergone a CS without complications to be healthy (Lundgren et al., 2015), with an assumption that these women will have a planned VBAC in their subsequent birth after CS (Lundgren et al., 2015). This common approach towards VBAC among midwives and obstetricians was problematic for the women in our study, because their desire to talk about the previous CS during the forthcoming VBAC was ignored. As a consequence, the women are not scheduled for more visits to the midwife at the antenatal clinic than other multiparous women. This was a cause of frustration for the women in our study who felt that the time was too limited. Although believing in and being determined to attempt a VBAC, they desired support to prepare themselves. Such preparation should involve working through their experiences of the CS and discussing possible complications due to the scar, types of pain relief that would not prolong labour, different types of prophylaxis and being involved in writing a birth plan. When no such support was given by the midwife, the women felt alone with their emotions and reflections, which also are described by other studies (Lundgren et al., 2012; Nilsson et al., 2015). Another explanation to why the women seems to be left without support could be that VBAC exists between the fields of midwifery and obstetric care, i.e. normality and pathology (Downe, 2008). The women identified themselves both as having the surgical scar from CS, and the striving for being like other women when wanting to give birth vaginally. The professionals seem not to understand this dilemma for the women.

The desire and determination to experience a VBAC and give birth like most women were apparent among the women in our study as well as in studies from countries with low VBAC rates (Emmett, Shaw, Montgomery, & Murphy, 2006; Fenwick et al., 2007; Meddings et al., 2007). In our study, memories of long-term consequences as a result of the scar, and concerns about not being able to take care of their older child after a second CS, similar to the findings in studies on women’s views of VBAC in countries where VBAC was less common (Fenwick et al., 2007).

The women in our study wanted more support in handling anxiety and fear of complications due to the previous CS. When the women’s anxiety was not alleviated, and their reflections not adequately discussed, they sought information about VBAC online, which has also been reported in other studies (Dahlen & Homer, 2013; Farnworth & Pearson, 2007; Fenwick, Gamble, & Mawson, 2003; Moffat et al., 2007). The women in our study told that the information on the internet was mostly negative highlighting the risks associated with VBAC, which contributed to even greater anxiety and worry. But interestingly, this information did not affect their determination for wanting a VBAC.

In our study, the women required support during VBAC because they wanted to experience different labour and birth than that of their first. They used various strategies to increase their chances of having a successful VBAC. The women used the previous CS as a reference when planning their VBAC; they were more focused, more physically active, demanded more of themselves and of the midwives all together taking greater responsibility for the birth. Although the women were confident about having a successful VBAC, they needed the support provided by the midwives. Similar findings have also been found by Godden et al. (2012) in a study from Australia investigating women’s perception of factors for having a successful VBAC (Godden et al., 2012). The women described that they had a desire for a natural birth, preparing for birth; meaning doing research and being armed with evidence, maternal actions, having support to have a VBAC and the importance of knowing other women who had a VBAC. It was also important with support from health professionals and a positive attitude among the staff towards VBAC (Godden et al., 2012).

The importance of midwives’ support for women’s positive birth experience has also been demonstrated in other studies (Berg, Lundgren, Hermansson, & Wahlberg, 1996; Halldorsdottir & Karlsdottir, 2011; Kennedy, 2000). The need to be viewed as an individual can be met through the affirmation of and familiarity with the midwife (Halldorsdottir & Karlsdottir, 2011), with whom a trusting relationship can be created through good communication and competent behaviour. By providing a sense of control, the midwife can support and guide the woman on her own terms but most importantly, the woman must feel that the midwife is present (Berg et al., 1996). Lack of support from midwives during labour can have a negative effect on women regardless of the mode of birth (Bohren, Hofmeyr, Sakala, Fukuzawa, & Cuthbert, 2017; Eliasson, Kainz, & von Post, 2008). In its complexity, childbirth can be both empowering and traumatic. Negative birth experiences might lead to fear of forthcoming births. In a study by Nilsson, Robertson, and Lundgren (2012) the aim was to describe the meaning of fear of childbirth from a long-term perspective it was found that every childbirth remains within the woman through her life. Therefore, it is important to help these women handle their experiences (Nilsson et al., 2012).

According to the women in our study, the experience of VBAC was dominantly described in terms of feelings of strength. They associated the experience of vaginal birth with what it means to be a woman, findings similar to other studies (Fenwick et al., 2007; Keedle, Schmied, Burns, & Dahlen, 2017; McGrath, Phillips, & Vaughan, 2010; Phillips et al., 2009). Achieving a natural birth was viewed as a significant aspect of their femininity and a major life event (Phillips et al., 2009). The women in a study by Fenwick et al. (2007) had strong views about the importance of cooperating with their bodies to achieve a VBAC and considered this an integral part of being a woman and mother. They also wanted control over the birthing experience, which they felt was lacking in their previous CS birth (Fenwick et al., 2007). In conclusion, birth is an important life event of significance for human beings (McGrath et al., 2010).

It was evident in our study that the experience of both CS and VBAC remained within the women as contrasting and comparing memories of two different births. This can be related to the findings in other studies, when concluding that women do not forget their childbirth experiences in a long-term perspective (Nilsson et al., 2012; Simkin, 1996).

The women described that although they had pain after the VBAC birth, it was considerably less than the painful healing process experienced after the CS. This finding is also confirmed in other studies (Emmett et al., 2006; Fenwick et al., 2007; Hill-Karbowski, 2014; Meddings et al., 2007). The VBAC enabled the women in our study to return to normal life afterwards which also have been described by other researchers (Emmett et al., 2006; Fenwick et al., 2007).

Another important emotional difference between vaginal birth and CS mentioned by the women in our study was not being separated from the baby immediately afterbirth. The women could start to connect with and breastfeed the baby with no delay. Some of the women in our study reflected upon whether the VBAC promoted the relationship with the baby to a greater extent than the CS. Vaginal birth is believed to provide the early opportunity to connect with the child (Fenwick et al., 2007; Meddings et al., 2007). Many women have commented on the distress of being separated from their baby after having a CS (Fenwick et al., 2003). In the study by Meddings et al. (2007), the issue of establishing the mother–infant relationship was frequently raised. Women were concerned about how different modes of birth affected the process, and their experiences did not depend solely on whether or not the birth was vaginal. Instead, mental clarity was perceived as important for the first mother–infant encounter, and women expressed less satisfaction when their senses were impaired by analgesia or anaesthesia (Meddings et al., 2007). However, the women in our study nuanced this by saying that they were more experienced as mothers when they gave birth to their second child.

The women in our study described what it means to be part of the birthing culture in Sweden, where it is common to give birth vaginally after a precious CS. Lundgren et al. (2015) studied midwives and obstetricians views on factors for improving the rate of VBAC in countries with high VBAC rates (including Sweden). The results can be interpreted as confirming the existing birthing culture in Sweden regarding VBAC; the clinicians regarded VBAC as the first option (Lundgren et al., 2015).

The results of our study have demonstrated that, for these women, VBAC is meaningful, important and special. Despite living in Sweden, which is regarded as a “VBAC friendly” country, it was difficult for the women to obtain information and support when having a VBAC, similar to findings from studies conducted in countries that have a low VBAC rate (Lundgren et al., 2012; Meddings et al., 2007; York, Briscoe, Walkinshaw, & Lavender, 2005). This knowledge has the potential to improve practice by highlighting the need for the adoption of a woman-centred care model (Berg, Asta Olafsdottir, & Lundgren, 2012) to guide midwives care of women who experienced CS. More research is needed about what type of information and support these women would benefit from after the CS, during the subsequent pregnancy and during the VBAC.

### Methodological reflections

To describe the phenomenon, women’s experiences of VBAC, a reflective lifeworld method based on phenomenological philosophy was used for this study (Dahlberg et al., 2008). The timing of the interviews was chosen in order to gain descriptions that were reflected upon; therefore, we decided to focus on the women’s long-term experience. The women provided rich and nuanced descriptions of their experiences during the interviews, helping us to gain a deeper understanding of the phenomenon being studied. However, the exclusion of women with psychiatric disease or women with experiences of fetal death or illness can have entailed a weaker variation of meanings to data. The analysis followed the principles for descriptive phenomenology as suggested by Dahlberg et al. (2008). During the research process, it is important, “not to take the indefinite as definite” (Dahlberg & Dahlberg, 2003). This means being aware of one’s pre-understanding of a phenomenon. For the first author, a midwife working at a labour ward, it was demanding. In order to decrease the influences of pre-understanding, she tried to make explicit her inner thoughts and experiences about the phenomenon during discussions with the co-authors. Another important principle for the analysis was to stay as long as possible in the analysis phase, i.e., moving back and forth (between a specific meaning unit to the sense of the whole interview) several times in order to minimize the risk of misunderstandings to avoid explicit interpretations. By doing so, the phenomenon was understood as a figure against a background or the reverse. This process is crucial for the trustworthiness of the findings in the study. After several reflective discussions during the whole process within the research group, we agreed on the findings.

For transferability, it is important to consider the context of this study. This study was conducted with a small group of Swedish-speaking women in one district of Sweden approximately one year after their VBAC experience. The women in our study wished for a VBAC for their subsequent birth. The context can also be understood as one where women are not free to make their own decision about the mode of birth and in which midwives’ cares for women, who have had a previous uncomplicated CS, during both pregnancy and birth. The fact that the findings are contextual, i.e. depending on time and place, does not mean that they have no meaning for other contexts but must be interpreted and related to the new context when transferred (Dahlberg et al., 2008). The findings can help to develop a more evidence-based professional approach when caring for women who have experienced a CS, during subsequent pregnancy and birth.

## Conclusions and clinical implications

The main finding in this study demonstrates that the essential meaning of women’s lived experience of VBAC is described as “to challenge oneself as a childbearing woman”, and can be further described by its four constituents: striving for support from professionals, desiring the experience, contrasting and comparing memories of two different births and being part of the birthing culture. Sweden is a country where VBAC is common; it is assumed that women with a previous uncomplicated CS will have a planned VBAC in their subsequent pregnancy. Surprisingly, our results revealed that the women lacked follow-up and support after the CS, during the subsequent pregnancy and the forthcoming VBAC. Women and their partners might benefit from being offered follow-up at an individual level. For these women, having a VBAC meant being capable of giving birth, regaining trust in their body and being able to give birth in the same way as most women. Enhanced support could be a key factor in helping women meeting the challenge and feel confident about giving birth vaginally despite their previous experiences of CS.

## References

[CIT0001] ACOG (2017). Practice bulletin No. 184: Vaginal birth after cesarean delivery. *Obstetrics and Gynecology*, 130(5), e217–13. 29064970 10.1097/AOG.0000000000002398

[CIT0002] Bengtsson J. (1995). Edmund Husserl: Fenomenologins idé: Daidalos Göteborg.

[CIT0003] Berg M., Asta Olafsdottir O., & Lundgren I. (2012). A midwifery model of woman-centred childbirth care–In Swedish and Icelandic settings. *Sexual & Reproductive Healthcare*, 3(2), 79–87. Retrieved from http://www.srhcjournal.org/article/S1877-5756(12)00018-3/abstract 22578755 10.1016/j.srhc.2012.03.001

[CIT0004] Berg M., Lundgren I., Hermansson E., & Wahlberg V. (1996). Women‘s experience of the encounter with the midwife during childbirth. *Midwifery*, 12(1), 11–15. 8715931 10.1016/s0266-6138(96)90033-9

[CIT0005] Betrán A., Temmerman M., Kingdon C., Mohiddin A., Opiyo N., Torloni M. R., … Downe S. (2018). Interventions to reduce unnecessary caesarean sections in healthy women and babies. *The Lancet*, 392(10155), 1358–1368. Retrieved from https://www.thelancet.com/journals/lancet/article/PIIS0140-6736(18)31927-5/fulltext 10.1016/S0140-6736(18)31927-5 30322586

[CIT0006] Bohren M. A., Hofmeyr G. J., Sakala C., Fukuzawa R. K., & Cuthbert A. (2017). Continuous support for women during childbirth. *Cochrane Database of Systematic Reviews*, (7). doi:10.1002/14651858.CD003766.pub6 PMC6483123 28681500

[CIT0007] Dahlberg H., & Dahlberg K. (2003). To not make definite what is indefinite: A phenomenological analysis of perception and its epistemological consequences in human science research. *The Humanistic Psychologist*, 31(4), 34–50.

[CIT0008] Dahlberg K. (2001). *Reflective lifeworld research*. Lund: Studentlitteratur.

[CIT0009] Dahlberg K. (2011). Lifeworld phenomenology for caring and health care research In Thomson G., Dykes F., & Downe S. (Eds.), *Qualitative research in midwifery and childbirth phenomenological approaches* (pp. 19–34). Abingdon, Oxon: Routledge.

[CIT0010] Dahlberg K., Dahlberg H., & Nyström M. (2008). *Reflective lifeworld research*. Lund: Studentlitteratur.

[CIT0011] Dahlberg K., Gjengedal E., & Råheim M. (2010). Editorial. *International Journal of Qualitative Studies on Health and Well-Being*, 5(4). Retrieved from https://www.tandfonline.com/doi/pdf/10.3402/qhw.v5i4.5800?needAccess=true 10.3402/qhw.v5i4.5800 PMC3014050 21203350

[CIT0012] Dahlen H. G., & Homer C. S. (2013). ‘Motherbirth or childbirth’? A prospective analysis of vaginal birth after caesarean blogs. *Midwifery*, 29(2), 167–173. Retrieved from http://ac.els-cdn.com/S0266613811001860/1-s2.0-S0266613811001860-main.pdf 22169525 10.1016/j.midw.2011.11.007

[CIT0013] Dencker A., Nilsson C., Begley C., Jangsten E., Mollberg M., Patel H., … Sparud-Lundin C. (2018). Causes and outcomes in studies of fear of childbirth: A systematic review. *Women and Birth*. doi:10.1016/j.wombi.2018.07.004 30115515

[CIT0014] Downe S. (2008). *Normal childbirth: Evidence and debate* (2. uppl. ed.). Edinburgh, New York: Churchill Livingstone/Elsevier.

[CIT0015] Eliasson M., Kainz G., & von Post I. (2008). Uncaring midwives. *Nursing Ethics*, 15(4), 500–511. Retrieved from http://nej.sagepub.com/content/15/4/500.full.pdf 18515439 10.1177/0969733008090521

[CIT0016] Elmir R., Schmied V., Wilkes L., & Jackson D. (2010). *Women’s perceptions and experiences of a traumatic birth: A meta‐ethnography* (Vol. 66, pp. 2142–2153). Oxford, UK: JAN (Journal of Advanced Nursing). 10.1111/j.1365-2648.2010.05391.x 20636467

[CIT0017] Emmett C. L., Shaw A. R., Montgomery A. A., & Murphy D. J. (2006). Women‘s experience of decision making about mode of delivery after a previous caesarean section: The role of health professionals and information about health risks. *BJOG: An International Journal of Obstetrics and Gynaecology*, 113(12), 1438–1445. Retrieved from http://onlinelibrary.wiley.com/store/10.1111/j.1471-0528.2006.01112.x/asset/j.1471-0528.2006.01112.x.pdf 17081180 10.1111/j.1471-0528.2006.01112.x

[CIT0018] EURO-PERISTAT (2008, 9 28). *European perinatal health report*. Retrieved from http://www.europeristat.com/reports/european-perinatal-health-report2008.html.

[CIT0019] EURO-PERISTAT (2013). *European perinatal health report: the health and care of pregnant women and babies in Europe in 2010*. Retrieved from http://www.europeristat.com/reports/european-perinatal-health-report-2010.html. 10.1136/jech.2009.087296 19679713

[CIT0020] Farnworth A., & Pearson P. H. (2007). Choosing mode of delivery after previous caesarean birth. *British Journal of Midwifery*, 15(4), 188–194. Retrieved from http://search.ebscohost.com/login.aspx?direct=true&db=c8h&AN=106143652&site=ehost-live

[CIT0021] Farnworth A., Robson S. C., Thomson R. G., Watson D. B., & Murtagh M. J. (2008). Decision support for women choosing mode of delivery after a previous caesarean section: A developmental study. *Patient Education and Counseling*, 71(1), 116–124. Retrieved from http://ac.els-cdn.com/S0738399107004806/1-s2.0-S0738399107004806-main.pdf 18255248 10.1016/j.pec.2007.11.020

[CIT0022] Fenwick J., Gamble J., & Hauck Y. (2007). Believing in birth–Choosing VBAC: The childbirth expectations of a self-selected cohort of Australian women. *Journal of Clinical Nursing*, 16(8), 1561–1570. Retrieved from http://onlinelibrary.wiley.com/store/10.1111/j.1365-2702.2006.01747.x/asset/j.1365-2702.2006.01747.x.pdf 17655545 10.1111/j.1365-2702.2006.01747.x

[CIT0023] Fenwick J., Gamble J., & Mawson J. (2003). Women‘s experiences of Caesarean section and vaginal birth after Caesarian: A Birthrites initiative. *International Journal of Nursing Practice*, 9(1), 10–17. Retrieved from http://onlinelibrary.wiley.com/store/10.1046/j.1440-172X.2003.00397.x/asset/j.1440-172X.2003.00397.x.pdf 12588615

[CIT0024] Frost J., Shaw A., Montgomery A., & Murphy D. J. (2009). Women‘s views on the use of decision aids for decision making about the method of delivery following a previous caesarean section: Qualitative interview study. *BJOG: An International Journal of Obstetrics and Gynaecology*, 116(7), 896–905. Retrieved from http://onlinelibrary.wiley.com/store/10.1111/j.1471-0528.2009.02120.x 19385964 10.1111/j.1471-0528.2009.02120.x

[CIT0025] Garthus-Niegel S., Soest T., Vollrath M., & Eberhard-Gran M. (2013). The impact of subjective birth experiences on post-traumatic stress symptoms: A longitudinal study. *Archives of Women‘S Mental Health*, 16(1), 1–10. Retrieved from https://link.springer.com/article/10.1007%2Fs00737-012-0301-3 10.1007/s00737-012-0301-3 22940723

[CIT0026] Godden B., Hauck Y., Hardwick T., & Bayes S. (2012). Women’s perceptions of contributory factors for successful vaginal birth after cesarean. *International Journal of Childbirth*, 2(2), 96–106. Retrieved from http://www.ingentaconnect.com/content/springer/ijc/2012/00000002/00000002/art00004

[CIT0027] Guise J. M., Eden K., Emeis C., Denman M. A., Marshall N., Fu R. R., … McDonagh M. (2010). Vaginal birth after cesarean: New insights. *Evidence Report/Technology Assessment*, *155*(191), 1–397. PMC4781304 20629481

[CIT0028] Halldorsdottir S., & Karlsdottir S. I. (2011). The primacy of the good midwife in midwifery services: An evolving theory of professionalism in midwifery. *Scandinavian Journal of Caring Sciences*, 25(4), 806–817. Retrieved from http://onlinelibrary.wiley.com/store/10.1111/j.1471-6712.2011.00886.x/asset/j.1471-6712.2011.00886.x.pdf 21480938 10.1111/j.1471-6712.2011.00886.x

[CIT0029] Hill-Karbowski E. (2014). *A feminist perspective on listening to women: Birth stories of vaginal birth following previous cesarean delivery* (3619959 Ph.D.). Marquette University, Ann Arbor.

[CIT0030] Karlstrom A., Radestad I., Eriksson C., Rubertsson C., Nystedt A., & Hildingsson I. (2010). Cesarean section without medical reason, 1997 to 2006: A Swedish register study. *Birth*, 37(1), 11–20. 20402717 10.1111/j.1523-536X.2009.00373.x

[CIT0031] Keedle H., Schmied V., Burns E., & Dahlen H. G. (2017). The journey from pain to power: A meta-ethnography on women’s experiences of vaginal birth after caesarean. *Women and Birth*, 31(1), 69–79. 28655602 10.1016/j.wombi.2017.06.008

[CIT0032] Kennedy H. P. (2000). A model of exemplary midwifery practice: Results of a Delphi study. *The Journal of Midwifery & Women’s Health*, 45(1), 4–19. Retrieved from http://ac.els-cdn.com/S1526952399000185/1-s2.0-S1526952399000185-main.pdf 10.1016/s1526-9523(99)00018-5 10772731

[CIT0033] Larkin P., Begley C. M., & Devane D. (2009). Women‘s experiences of labour and birth: An evolutionary concept analysis. *Midwifery*, 25(2), e49–59. Retrieved from http://ac.els-cdn.com/S0266613807000824/1-s2.0-S0266613807000824-main.pdf 17996342 10.1016/j.midw.2007.07.010

[CIT0034] Larsson B., Hildingsson I., Ternström E., Rubertsson C., & Karlström A. (2018). Women‘s experience of midwife-led counselling and its influence on childbirth fear: A qualitative study. *Women and Birth: Journal of the Australian College of Midwives*. Retrieved from https://www.womenandbirth.org/article/S1871-5192(17)30328-1/fulltext 10.1016/j.wombi.2018.04.008 29709431

[CIT0035] Larsson B., Karlström A., Rubertsson C., & Hildingsson I. (2015). The effects of counseling on fear of childbirth. *Acta Obstetricia Et Gynecologica Scandinavica*, 94(6), 629–636. Retrieved from http://gothenburg.summon.serialssolutions.com/2.0.0/link/0/eLvHCX 25772528 10.1111/aogs.12634

[CIT0036] Lundgren I., Begley C., Gross M. M., & Bondas T. (2012). ‘Groping through the fog‘: A metasynthesis of women‘s experiences on VBAC (Vaginal birth after Caesarean section). *BMC Pregnancy and Childbirth*, 12, 85 Retrieved from http://www.ncbi.nlm.nih.gov/pmc/articles/PMC3506503/pdf/1471-2393-12-85.pdf 22909230 10.1186/1471-2393-12-85 PMC3506503

[CIT0037] Lundgren I., van Limbeek E., Vehvilainen-Julkunen K., & Nilsson C. (2015). Clinicians’ views of factors of importance for improving the rate of VBAC (vaginal birth after caesarean section): A qualitative study from countries with high VBAC rates. *BMC Pregnancy and Childbirth*, 15, 196 Retrieved from http://www.ncbi.nlm.nih.gov/pmc/articles/PMC4552403/pdf/12884_2015_Article_629.pdf 26314295 10.1186/s12884-015-0629-6 PMC4552403

[CIT0038] McGrath P., Phillips E., & Vaughan G. (2010). Vaginal birth after Caesarean risk decision-making: Australian findings on the mothers‘ perspective. *International Journal of Nursing Practice*, 16(3), 274–281. Retrieved from http://onlinelibrary.wiley.com/doi/10.1111/j.1440-172X.2010.01841.x/abstract 20618538 10.1111/j.1440-172X.2010.01841.x

[CIT0039] Meddings F., Phipps F. M., Haith-Cooper M., & Haigh J. (2007). Vaginal birth after caesarean section (VBAC): Exploring women‘s perceptions. *Journal of Clinical Nursing*, 16(1), 160–167. Retrieved from http://onlinelibrary.wiley.com/store/10.1111/j.1365-2702.2005.01496.x/asset/j.1365-2702.2005.01496.x.pdf 17181678 10.1111/j.1365-2702.2005.01496.x

[CIT0040] Moffat M. A., Bell J. S., Porter M. A., Lawton S., Hundley V., Danielian P., & Bhattacharya S. (2007). Decision making about mode of delivery among pregnant women who have previously had a caesarean section: A qualitative study. *BJOG: an International Journal of Obstetrics and Gynaecology*, 114(1), 86–93. Retrieved from http://onlinelibrary.wiley.com/store/10.1111/j.1471-0528.2006.01154.x/asset/j.1471-0528.2006.01154.x.pdf 17233863 10.1111/j.1471-0528.2006.01154.x

[CIT0041] Nilsson C., Hessman E., Sjöblom H., Dencker A., Jangsten E., Mollberg M., … Begley C. (2018). Definitions, measurements and prevalence of fear of childbirth:: A systematic review. *BMC Pregnancy and Childbirth*, 18(1). Retrieved from https://bmcpregnancychildbirth.biomedcentral.com/track/pdf/10.1186/s12884-018-1659-7 10.1186/s12884-018-1659-7 PMC5766978 29329526

[CIT0042] Nilsson C., Lalor J., Begley C., Carroll M., Gross M. M., Grylka-Baeschlin S., … Healy P. (2017). Vaginal birth after caesarean: Views of women from countries with low VBAC rates. *Women and Birth*, 30(6), 481–490. 10.1016/j.wombi.2017.04.009 28545775

[CIT0043] Nilsson C., Robertson E., & Lundgren I. (2012). An effort to make all the pieces come together: Women‘s long-term perspectives on their experiences of intense fear of childbirth. *International Journal of Childbirth*, 2(4), 255–268. Retrieved from http://www.ingentaconnect.com/content/springer/ijc/2012/00000002/00000004/art00007

[CIT0044] Nilsson C., van Limbeek E., Vehvilainen-Julkunen K., & Lundgren I. (2015). Vaginal birth after cesarean: Views of women from countries with high VBAC rates. *Qualitative Health Research*, 1–16. Retrieved from http://qhr.sagepub.com/content/early/2015/11/02/1049732315612041.abstract 10.1177/1049732315612041 26531882

[CIT0045] Nystedt A., & Hildingsson I. (2018). Women’s and men’s negative experience of child birth – A cross-sectional survey. *Women and Birth*, 31(2), 103–109. 28789827 10.1016/j.wombi.2017.07.002

[CIT0046] OECD (2017). *“Caesarean sections”, in Health at a Glance 2017: OECD Indicators*. Paris: Author.

[CIT0047] Phillips E., McGrath P., & Vaughan G. (2009). ‘I wanted desperately to have a natural birth‘: Mothers‘ insights on vaginal birth after Caesarean (VBAC). *Contemporary Nurse*, 34(1), 77–84. 20230174 10.5172/conu.2009.34.1.077

[CIT0048] Poignant M., Hjelmstedt A., & Ekeus C. (2012). Indications for operative delivery between 1999–2010 and induction of labor and epidural analgesia on the risk of operative delivery–A population based Swedish register study. *Sexual & Reproductive Healthcare*, 3(4), 129–134. Retrieved from http://ac.els-cdn.com/S1877575612000511/1-s2.0-S1877575612000511-main.pdf 23182444 10.1016/j.srhc.2012.10.004

[CIT0049] Rijnders M., Baston H., Schönbeck Y., Van Der Pal K., Prins M., Green J., & Buitendijk S. (2008). Perinatal factors related to negative or positive recall of birth experience in women 3 years postpartum in the Netherlands. *Birth*, 35(2), 107–116. Retrieved from https://onlinelibrary.wiley.com/doi/abs/10.1111/j.1523-536X.2008.00223.x 18507581 10.1111/j.1523-536X.2008.00223.x

[CIT0050] Ryding E. L., Persson A., Onell C., & Kvist L. (2003). An evaluation of midwives‘ counseling of pregnant women in fear of childbirth. *Acta Obstetricia Et Gynecologica Scandinavica*, 82(1), 10–17. Retrieved from http://onlinelibrary.wiley.com/store/10.1034/j.1600-0412.2003.820102 12580833 10.1034/j.1600-0412.2003.820102.x

[CIT0051] SBF (2018). *Svenska barnmorskeförbundet (The Swedish Association of Midwives) Kompetensbeskrivning för legitimerad barnmorska (Descriptions of competencies for midwives)*. Stockholm, Sweden: The Swedish Association of Midwives.

[CIT0052] Simkin P. (1991). Just another day in a woman’s life? Women’s long‐term perceptions of their first birth experience. Part I. *Birth*, 18(4), 203–210. Retrieved from https://onlinelibrary.wiley.com/doi/pdf/10.1111/j.1523-536X.1991.tb00103.x 1764149 10.1111/j.1523-536x.1991.tb00103.x

[CIT0053] Simkin P. (1996). The experience of maternity in a woman‘s life. *Journal of Obstetric, Gynecologic & Neonatal Nursing*, 25(3), 247–252. Retrieved from http://onlinelibrary.wiley.com/store/10.1111/j.1552-6909.1996.tb0243 10.1111/j.1552-6909.1996.tb02432.x 8683360

[CIT0054] Smarandache A., Kim T. H. M., Bohr Y., & Tamim H. (2016). Predictors of a negative labour and birth experience based on a national survey of Canadian women. *BMC Pregnancy and Childbirth*, 16(1). Retrieved from https://bmcpregnancychildbirth.biomedcentral.com/track/pdf/10.1186/s12884-016-0903-2 10.1186/s12884-016-0903-2 PMC4870779 27193995

[CIT0055] Socialstyrelsen (2017). (National Board of Health and Welfare) Graviditeter, förlossningar och nyfödda barn-Medicinska födelseregistret. Pregnancies, Deliveries and Newborn infants – The Swedish Medical Birth Register. Retrieved from http://www.socialstyrelsen.se/Lists/Artikelkatalog/Attachments/20807/2018-1-6.pdf.

[CIT0056] Waldenström U., Hildingsson I., Rubertsson C., & Rådestad I. (2004). A negative birth experience: prevalence and risk factors in a national sample. *Birth*, 31(1), 17–27. Retrieved from https://onlinelibrary.wiley.com/doi/abs/10.1111/j.0730-7659.2004.0270.x 15015989 10.1111/j.0730-7659.2004.0270.x

[CIT0057] Wang H. H., Chung U. L., Sung M. S., & Wu S. M. (2006). Development of a web-based childbirth education program for vaginal birth after C-section (VBAC) mothers. *Journal of Nursing Research*, 14(1), 1–8. 16547901 10.1097/01.jnr.0000387557.19071.b3

[CIT0058] Wiklund I., Andolf E., Lilja H., & Hildingsson I. (2012). Indications for cesarean section on maternal request – Guidelines for counseling and treatment. *Sexual & Reproductive Healthcare*, 3(3), 99–106. Retrieved from http://ac.els-cdn.com/S1877575612000304/1-s2.0-S1877575612000304-main.pdf 22980734 10.1016/j.srhc.2012.06.003

[CIT0059] Wulcan A.-C., & Nilsson C. (2019). Midwives’ counselling of women at specialised fear of childbirth clinics: A qualitative study. *Sexual & Reproductive Healthcare*, 19, 24–30. Retrieved from https://ac.els-cdn.com/S1877575618301095/1-s2.0-S1877575618301095-main.pdf 30928131 10.1016/j.srhc.2018.12.001

[CIT0060] York S., Briscoe L., Walkinshaw S., & Lavender T. (2005). Why women choose to have a repeat caesarean section. *British Journal of Midwifery*, 13(7), 440–445. Retrieved from http://search.ebscohost.com/login.aspx?direct=true&db=c8h&AN=106524713&site=ehost-live

